# Morphological and Biological Characteristics of *Staphylococcus aureus* Biofilm Formed in the Presence of Plasma

**DOI:** 10.1089/mdr.2019.0068

**Published:** 2019-05-30

**Authors:** Ayami Sato, Tetsuo Yamaguchi, Masakaze Hamada, Daisuke Ono, Shiro Sonoda, Takashi Oshiro, Makoto Nagashima, Keisuke Kato, Shinichi Okazumi, Ryoji Katoh, Yoshikazu Ishii, Kazuhiro Tateda

**Affiliations:** ^1^ Department of Microbiology and Infectious Diseases, Faculty of Medicine, Toho University, Tokyo, Japan.; ^2^ Department of Surgery, Graduate School of Medicine, Toho University, Chiba, Japan.; ^3^ Department of Surgery, Sakura Medical Center, Toho University, Chiba, Japan.; ^4^ The Integrated Pulmonology, Tokyo Medical and Dental University, Tokyo, Japan.; ^5^ Department of Pharmacology, Faculty of Pharmaceutical Sciences, Toho University, Chiba, Japan.

**Keywords:** MRSA, plasma biofilm, COCRM, *Staphylococcus aureus*, CA-MRSA

## Abstract

Characteristics of *Staphylococcus aureus* infections include biofilm formation, leading to the spread of bacteria to the bloodstream causing sepsis and metastatic infections. In particular, in methicillin-resistant *S. aureus* (MRSA) infections, biofilm formation critically hampers treatment and causes poor prognosis. We explored the biofilm formation of MRSA in the presence or absence of plasma and compared morphological characteristics, accumulation of antibiotics, and resistance to bactericidal activity, using continuous optimizing confocal reflection microscopy. Addition of plasma significantly increased biofilm formation, which is characterized by an uneven surface and aggregation of bacteria (hereafter plasma biofilm). The flow-cell system, which enabled a continuous supply of plasma, accelerated biofilm formation in both the tested strains of MRSA (BAA1556 and N315). Accumulation of green fluorescence-labeled vancomycin was observed within 5 minutes in the plasma-free biofilm, but not in the plasma biofilm. Delay of accumulation was also observed for daptomycin in plasma biofilm. Plasma biofilm bacteria were more resistant to anti-MRSA antibiotics than plasma-free biofilm bacteria. These data demonstrate that the plasma biofilm of *S. aureus* is substantially different from the plasma-free biofilm. Plasma biofilm, especially in the flow-cell system, could be a clinically relevant model to analyze MRSA infections and treatment.

## Introduction

*S**taphylococcus aureus* is a gram-positive coccus that colonizes the nasal mucosa and skin of healthy individuals.^1^ This organism can cause a wide range of diseases from skin or soft tissue infections to systemic and fatal diseases.^1–3^ In particular, methicillin-resistant *S. aureus* (MRSA) is a hazardous organism because of its resistance to multiple antibiotics and biofilm formation ability. Device- or focal infection site-derived blood-stream infections are examples of severe cases of MRSA diseases. The appearance of community-acquired MRSA (CA-MRSA), in addition to the conventional hospital-acquired MRSA (HA-MRSA), poses a high risk to immune compromised patients and healthy individuals.

It is well known that biofilm-forming bacteria can survive in the presence of high concentrations of antimicrobial agents.^4^ Bacterial biofilms consist of a variety of components and substances from both the bacteria (polysaccharide, peptidoglycan, and DNA) and the host (cell debris, coagulation products, and DNA). Biofilm composition varies depending on the causative organism and/or host factors.

*S. aureus* possesses a specific virulence factor called coagulase, which plays a significant role in biofilm formation during *S. aureus* infections. Coagulase binds to host prothrombin and forms active staphylothrombin complexes, which converts soluble monomeric fibrinogen into self-polymerizing insoluble fibrin and activates a coagulation cascade.^5^ This phenomenon has been used in clinical microbiology laboratories as the coagulase test for the identification of *Staphylococcus* species. In recent years, some reports have suggested that *S. aureus* utilize the fibrin and fibrinogen recruited by coagulase to form the biofilm scaffold.^6^

Previous reports demonstrated mechanisms of antibiotic resistance in biofilm-forming bacteria. Even in plasma-free conditions, biofilm-forming bacteria exhibit high resistance to a variety of antibiotics. The barrier to antibiotic accumulation and penetration due to the biofilm structure, in addition to the tolerant and static nature of bacteria in biofilms, are crucial for antibiotic resistance in biofilm-forming organisms. However, knowledge about comparative antibiotic resistance in biofilms with or without plasma is limited.^7,8^

In this study, we compared the characteristics of *S. aureus* biofilm formed in the presence or absence of plasma. The analysis was made on two reference strains that were representatives of CA-MRSA and HA-MRSA, respectively. We applied continuous optimizing confocal reflection microscopy (COCRM)^9^ to observe morphological characteristics of the biofilm. COCRM enabled us to continuously observe the same biofilm without adding fluorescent dyes. Finally, antibiotic resistance was compared in *S. aureus* biofilms formed with plasma-free and plasma-added conditions.

## Materials and Methods

### Bacterial strains and culture conditions

Two strains of MRSA, ATCC BAA1556 (FPR3757 strain; USA300 clone) and N315 (New York/Japan clone), were used in this study.^10,11^ These strains were stored in Brain Heart Infusion (BHI) broth (Becton Dickinson) with 20% glycerol at −80°C. Before use, the strains were grown overnight on BHI agar and then subcultured for 12 hours in BHI broth at 35°C with shaking at 160 rpm under aerobic conditions. One milliliter of the culture was centrifuged at 9,000 × *g*, and the bacterial pellets were suspended in Tryptic Soy Broth (Becton Dickinson) containing 0.25% glucose (TSBG). This step was repeated once again. The minimum inhibitory concentrations (MICs) of vancomycin, linezolid, and rifampicin for both strains were 0.5, 1, and 0.008 μg/mL, respectively. The MICs of daptomycin for BAA1556 and N315 were 0.5 and 0.25 μg/mL, respectively. Pathogen protocols were approved by Toho University Safety Committee for Pathogen (approval no. 17-55-58).

### Quantification of biofilm formation in 96-well plate using crystal violet staining method

Rabbit plasma (Eiken Chemical, Tokyo, Japan) was dissolved in TSBG and then diluted further with TSBG. Final plasma concentrations were adjusted to the range of 0–12.5% (v/v). Overnight bacterial cultures were diluted to an OD_600_ of 0.01 (10^6^–10^7^ CFU/mL) using TSBG containing plasma. One hundred microliter aliquots were then inoculated into individual wells of a 96-well round-bottom polystyrene plate and incubated at 35°C for 6–18 hours. After incubation, biofilms were quantified following a previously reported crystal violet staining method, except for the use of 33.3% acetic acid instead of ethanol.^12^

### Visualization of biofilm structure in a glass-based dish using COCRM

The overnight cultures were diluted to an OD_600_ of 0.01 using TSBG or TSBG containing 7.14% plasma. Two-milliliter aliquots were inoculated into glass-based dishes (IWAKI, Tokyo, Japan) and incubated at 35°C for 12 or 24 hours. After incubation, the biofilms formed on the glass surface were washed with water, and the biofilm structures were visualized by COCRM using a Carl Zeiss Laser Scanning Microscope (LSM 710) equipped with a 63 × /1.40 numerical aperture plan-apochromatic objective lens.^9,13^ Biofilms and the glass surfaces were illuminated with 514 nm argon laser, and the reflected light was collected through a 505–530 nm band-pass filter. As a beam splitter, an NT 80/20 half mirror was used. COCRM images were analyzed with Carl Zeiss software (ZEN 2011).

### Biofilm formation in a flow-cell system

Flow cells (IBI Scientific, Dubuque, IA) were continuously irrigated with fresh medium (TSBG or TSBG containing 0.39% plasma) using a peristaltic pump (Ismatec, Glattbrugg, Switzerland).^14^ The overnight bacterial cultures were diluted to an OD_600_ of 0.01 using TSBG. Four hundred microliter aliquots were then injected from each of the three-way stopcocks connected to the distal ends of the flow cells and cultured for 1 hour at 35°C on the glass surface of the flow cells without flow. After confirmation of initial adhesion using COCRM, each medium flowed at a rate of 0.2 mL/min. Biofilms were incubated for 24 hours at 35°C. Biofilm formation was evaluated by COCRM every 3 hours.

### Effects of medium changes on biofilm formation in a glass-based dish

We established a protocol similar to the flow cell model using glass dishes to analyze the antimicrobial activity against plasma biofilm, because the glass dish is very versatile to observe the plasma biofilm formation. Overnight cultures were diluted to an OD_600_ of 0.01 in TSBG or TSBG containing 0.78% plasma. Two-milliliter aliquots were inoculated into glass-based dishes and statically incubated at 35°C under aerobic conditions for 1 hour to enable initial adhesion. This step makes it possible to observe bacteria adhesion to the glass surface by confocal reflection microscopy (CRM). Subsequently, the culture media were refreshed every hour to maintain a constant plasma supply for 6 hours in total. The medium with plasma was replaced five times during 6 hours (at 1, 2, 3, 4, and 5 hours). After incubation, biofilms formed on the glass surface were washed with water, and the biofilm structures were visualized by COCRM.

### Visualization of antibiotic penetration and accumulation inside biofilm using fluorescent-labeled vancomycin and daptomycin

Overnight cultures were diluted to an OD_600_ of 0.01 in TSBG or TSBG containing 0.78% plasma. Two-milliliter aliquots were inoculated into glass-based dishes and incubated at 35°C. During 6 hours incubation, the culture media were refreshed every hour. After removing floating bacteria by washing twice with phosphate buffered saline (PBS), fluorescent-labeled antibiotics (BODIPY-FL-vancomycin or BODIPY-FL-daptomycin) were added to the glass-based dishes. BODIPY-FL-vancomycin was purchased from Invitrogen, while BODIPY-FL-daptomycin was synthesized from BODIPY-FL NHS Ester (Invitrogen) and daptomycin by Toho University School of Pharmaceutical Sciences. Both antibiotics were dissolved in dimethyl sulfoxide and then diluted with PBS having calcium ion concentration adjusted to 50 mg/L. The final concentration of the BODIPY-FL-vancomycin added to biofilms was 2 μg/mL. The synthesized BODIPY-FL-daptomycin showed weak fluorescence intensity compared to vancomycin, and therefore the final concentration was adjusted to 20 μg/mL. The permeability of both antibiotics inside biofilm structures was monitored for 60 minutes using a Carl Zeiss Laser Scanning Microscope (LSM 710). Biofilms were illuminated with 488 nm argon laser, and the reflected light was detected at a gain of 550. Likewise, the BODIPY-FL dyes were illuminated with 488 nm argon laser, and the green fluorescence was detected for vancomycin and daptomycin at a gain of 670 and 600, respectively. Serial images were analyzed with a Carl Zeiss software (ZEN 2011).

### Quantitative determination of viable bacterial number after exposure to antibiotics

The overnight cultures were diluted to an OD_600_ of 0.01 using TSBG or TSBG containing 0.78% plasma. Two-milliliter aliquots were inoculated into six-well polystyrene plates and incubated at 35°C. During 6 hours incubation, the culture medium was refreshed every hour. After the removal of floating bacteria, biofilms were washed twice with TSBG, and then treated with 64 × MIC of antibiotics for 12 hours at 35°C. These antibiotics were dissolved in TSBG with calcium concentration adjusted to 50 mg/L. Only rifampicin was dissolved in methanol and then diluted with TSBG containing 50 mg/L Ca^2+^. The final concentration of methanol was 500 μL/mL. In this experiment, TSBG containing 50 mg/L Ca^2+^ and methanol were used as broth control. Floating bacteria were removed after treatment, and the biofilms were washed twice with saline. Two milliliters of proteinase K (0.1 mg/mL; WAKO, Osaka, Japan) dissolved in PBS was used to loosen the biofilm structures. Proteinase K-treated biofilms were peeled off with a scraper.^15^ The biofilm suspensions were gently placed at 37°C for 30 minutes and then diluted with saline and plated onto nutrient agar to count the number of biofilm-embedded bacteria. After growth at 35°C, the colonies were counted.

### LIVE/DEAD bacterial viability assay

Viability staining of *S. aureus* biofilm with or without plasma after treatments with different anti-MRSA agents was performed. Overnight cultures were diluted to an OD_600_ of 0.01 in TSBG or TSBG containing 0.78% plasma. Then, 200 μL aliquots were inoculated into eight-well coverglass chamber (IWAKI) and incubated at 35°C. The media were refreshed every hour for 6 hours. After the removal of floating bacteria, biofilms were washed twice with TSBG, and then treated with 64 × MIC of antibiotics for 12 hours at 35°C. These antibiotics were dissolved in TSBG with Ca^2+^ concentration adjusted to 50 mg/L. Rifampicin was dissolved in methanol and then diluted with TSBG containing 50 mg/L. The final concentration of methanol was 500 μL/mL. In this experiment, TSBG containing 50 mg/L Ca^2+^ and methanol were used as broth control. Floating bacteria were removed after treatment. The bacterial viability in the biofilm was determined by FilmTracer™ LIVE/DEAD^®^ (Molecular Probes) Biofilm Viability Kit. Viable cells were stained with SYTO 9 (green), whereas dead bacteria were stained with propidium iodide (red). After treatment, suspended bacteria were removed and stained with LIVE/DEAD staining solution for 20 minutes. After staining, the supernatant was removed and washed twice with pure water, and then the bactericidal activity of the antibacterial agent was visually evaluated by confocal laser scanning microscope.

### Statistical analysis

**FIG. 1.**
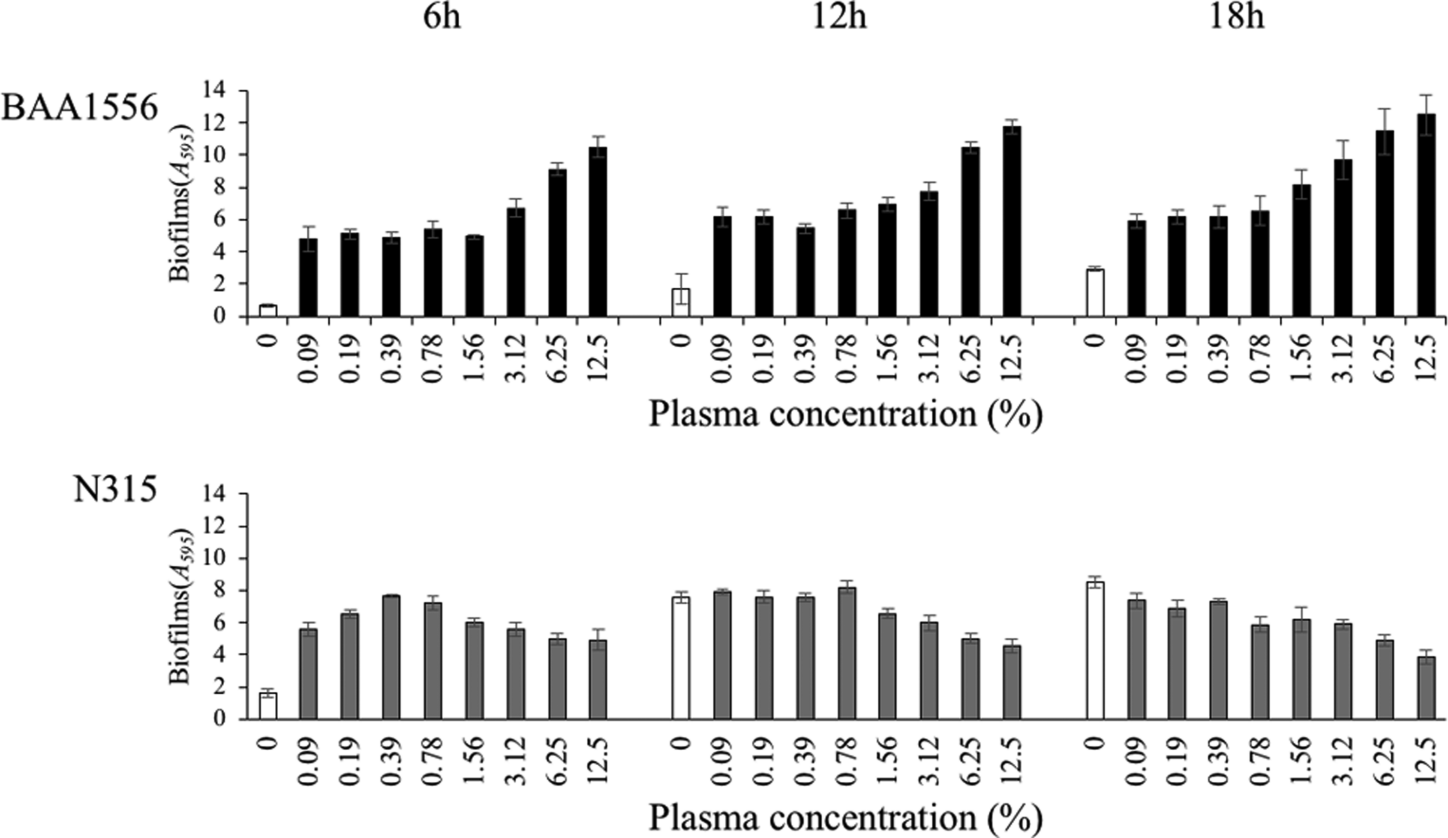
Effects of plasma on biofilm formation in *Staphylococcus aureus*. Mean biofilm biomass (±standard deviation, *n* = 4) of BAA1556 and N315 strains was measured by crystal violet staining in the presence of various concentrations of rabbit plasma for 6, 12, and 18 hours. Both strains showed a significant increase in biofilm formation at all plasma concentrations in 6 hours cultures (ANOVA, Tukey's test, *p* < 0.05). ANOVA, analysis of variance.

The results analyzed using GraphPad Prism 5.0 are presented as mean value ± standard deviation. Results were considered statistically significant when the *p* value was <0.05. The results of quantitative experiments performed in Figs. 1 and 6 were analyzed by analysis of variance (ANOVA) with a Tukey's test.

## Results

### Effects of plasma on *S. aureus* biofilm formation

We examined biofilm formation in various concentrations of plasma for two strains of *S. aureus*, BAA1556, and N315. In both strains, the presence of plasma even at low concentrations (0.09%) induced an increase in biofilm formation after 6 hours of incubation (Fig. 1). In the BAA1556 strain, concentration-dependent increase in biofilm formation was observed from 3.12% plasma, and substantial growth was observed at 12 and 18 hours. In the N315 strain, more than threefold increase in biofilm formation was observed from 6 to 12 hours in the absence of plasma, with no difference between 0% and 0.09% plasma at 12 hours. On the contrary, a trend of concentration-dependent gradual reduction in biofilm mass was observed in the N315 strain, from 0.78% or higher concentration of plasma.

### Morphological characteristics of *S. aureus* biofilm in the presence of plasma

**FIG. 2.**
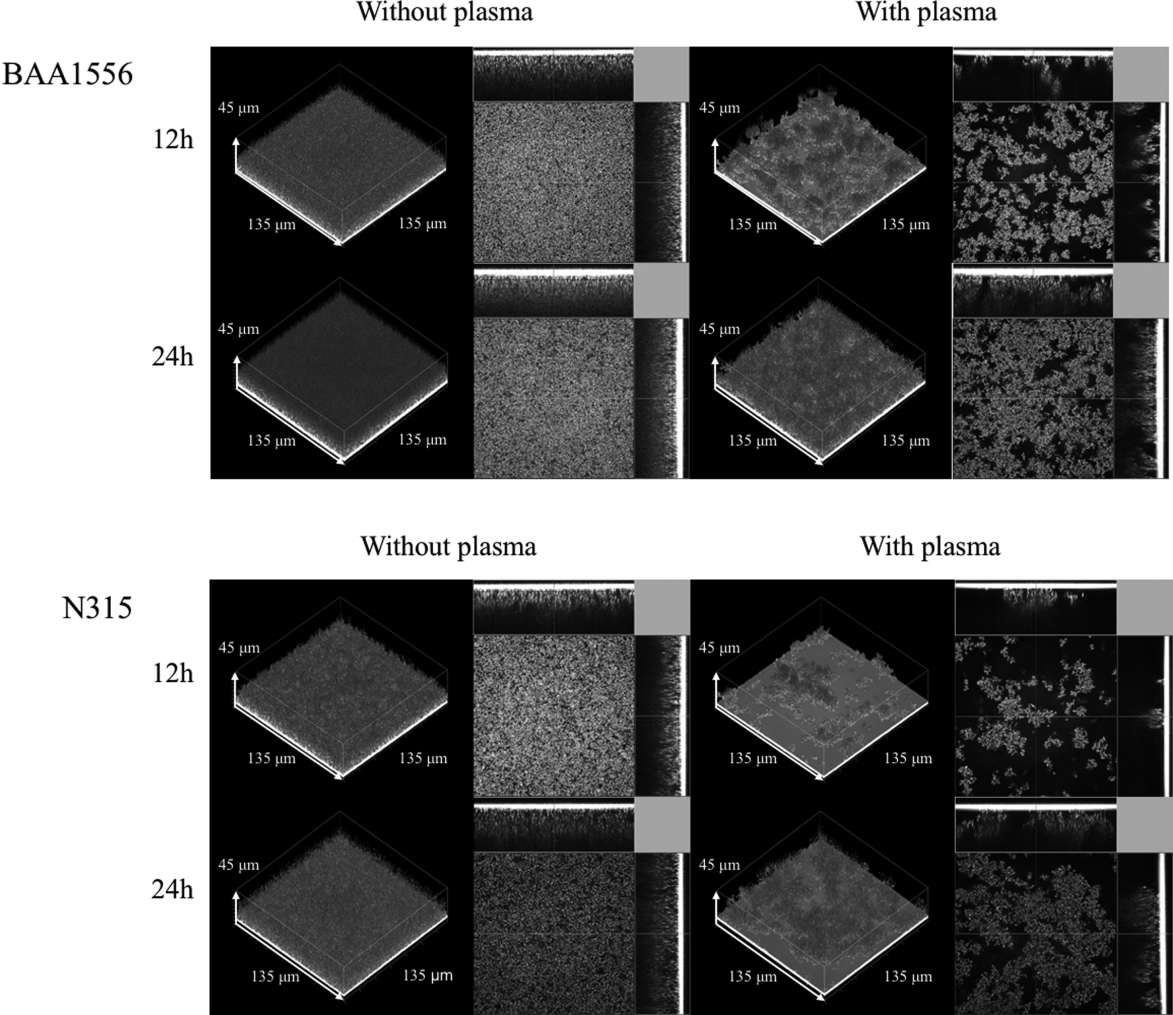
Morphological characteristics of *Staphylococcus aureus* biofilm in the presence of plasma. The biofilm formations of BAA1556 and N315 strains were examined by COCRM in the presence or absence of 7.14% plasma for 12 and 24 hours. Each projection shows a 3D image (135 × 135 × 45 μm; xyz) and the cross section of the biofilm. 3D, three dimensional; COCRM, continuous optimizing confocal reflection microscopy.

*S. aureus* biofilm was formed upon static incubation in the presence or absence of plasma, and the microstructure of the biofilm was compared by COCRM. As shown in Fig. 2, a uniform and flat biofilm was formed in the absence of plasma in both BAA1556 and N315 strains. In contrast, uneven and aggregate-structured biofilm was observed in the presence of plasma in both strains. The microscopic morphology of *S. aureus* biofilm formed in the presence of plasma was distinctly different from those formed without plasma, and therefore, these are hereafter referred to as plasma biofilm.

### *S. aureus* biofilm formation in the flow-cell condition in the presence of plasma

**FIG. 3.**
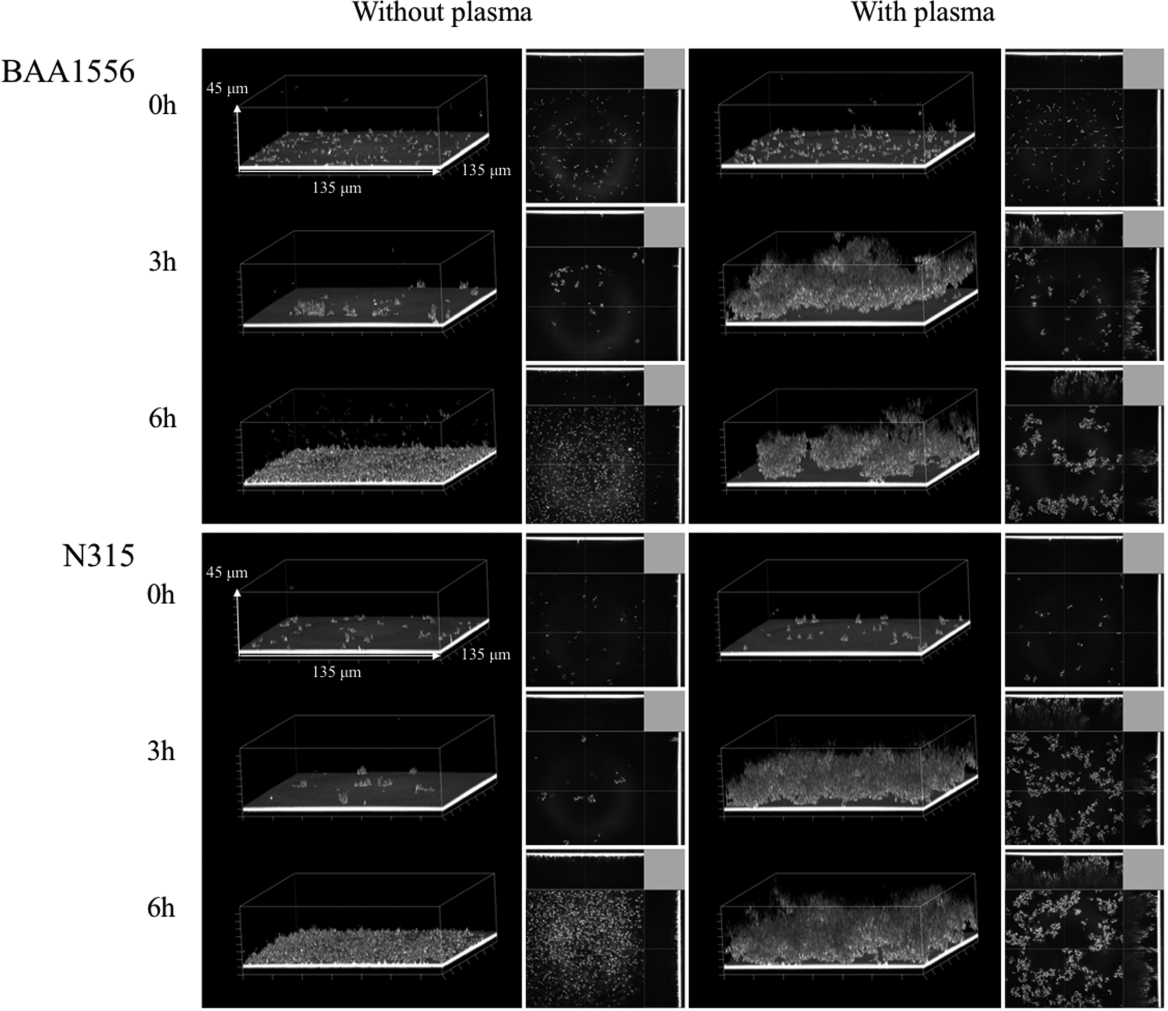
*Staphylococcus aureus* biofilm formed in a flow-cell condition in the presence of plasma. Biofilm formations of BAA1556 and N315 strains in the presence or absence of 0.39% plasma were examined by COCRM just after the start of the experiment, and after 3 and 6 hours of incubation. Each projection shows a 3D image (135 × 135 × 45 μm; xyz) and the cross section of the biofilm.

The flow-cell system, which represents a clinically relevant condition, was used to observe the effect of continuous plasma supply on biofilm formation. We compared early time points of biofilm formation in the flow-cell in the presence or absence of plasma by COCRM (Fig. 3). Without plasma, attachment of few bacteria was observed after 3 hours of incubation. In contrast, aggregates of biofilm formation were observed in the presence of plasma in both *S. aureus* strains. The structure of this plasma biofilm after 3 hours in the flow-cell system was similar to the plasma biofilm in a static condition at 12 hours (Fig. 2). However, in the flow-cell condition, the detachment of aggregated plasma biofilm was seen at later time points of incubation (data not shown).

### Penetration and accumulation of vancomycin in *S. aureus* biofilm

**FIG. 4.**
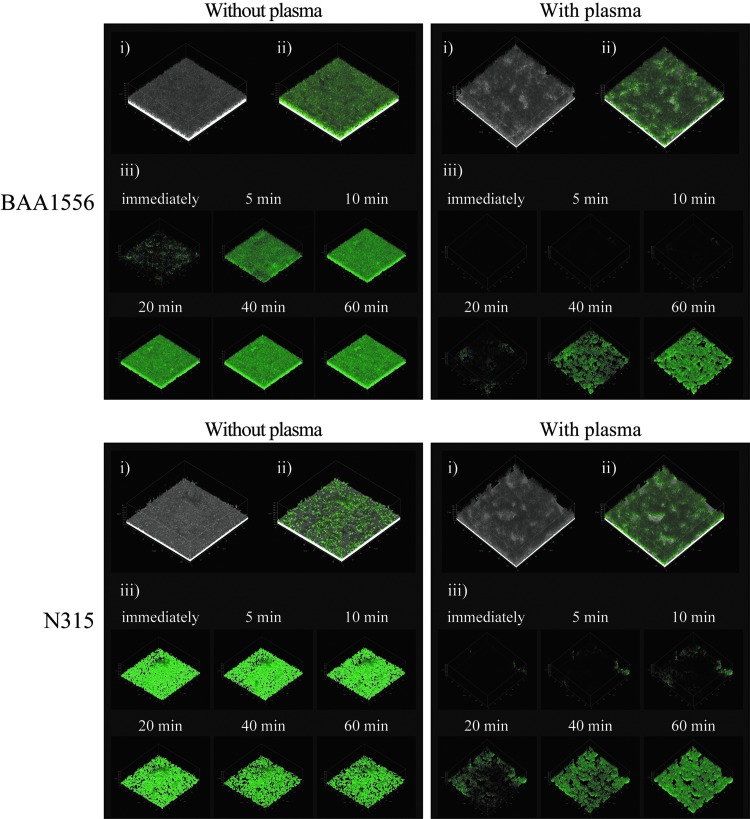
Accumulation of vancomycin in biofilm formed in the presence or absence of plasma. Biofilms of BAA1556 and N315 strains were formed during 6 hours incubation with media refreshed every hour in the presence or absence of 0.78% plasma. Then, green fluorescence-labeled vancomycin was added and incubated for 60 minutes. Penetration and accumulation of vancomycin were compared immediately after addition of labeled vancomycin, 5, 10, 20, 40, and 60 minutes using CLSM. The methods of observation were as follows: (i) continuous optimizing confocal reflection microscopy (COCRM) images, (ii) imaging of biofilm at 60 minutes after treatment with BODIPY FL^®^ Vancomycin was performed by CLSM, (iii) only the *green color* was extracted and shown over time by CLSM. CLSM, confocal laser scanning microscopy.

The BAA1556 and N315 strains were incubated in TSBG medium in the presence or absence of 0.78% plasma. The culture medium was refreshed every hour for 6 hours. In the control condition, the biofilm structure with or without plasma was examined by COCRM (Fig. 4-i). Then, green fluorescence-labeled vancomycin was added to the culture, and the biofilm was observed for an additional 1 hour. The penetration and accumulation of vancomycin were compared in the presence or absence of plasma by confocal laser scanning microscopy. As expected, striking differences in biofilm structures were observed between the samples with or without plasma. The addition of green fluorescent vancomycin further emphasized the differences between the biofilms with or without plasma. In the plasma-free biofilm, the diffused distribution of green fluorescence was observed in both strains. In contrast, rough structures on the surface, irregularly stained with green fluorescence, were observed in the biofilm grown in the presence of plasma (Fig. 4-ii). In the *S. aureus* biofilm in plasma-free condition, diffusion and strong green fluorescence were observed 5 minutes after administration of vancomycin, which intensified at later time points. In contrast, plasma biofilm exhibited different staining characteristics in both strains of *S. aureus*. Green fluorescence was faintly observed in plasma biofilm at 5 minutes, and thereafter accumulation of green fluorescence increased gradually. These data demonstrate a delay in penetration and accumulation of vancomycin in plasma biofilm, compared to plasma-free biofilm (Fig. 4-iii).

### Evaluation of accumulation of daptomycin in *S. aureus* biofilm over time

**FIG. 5.**
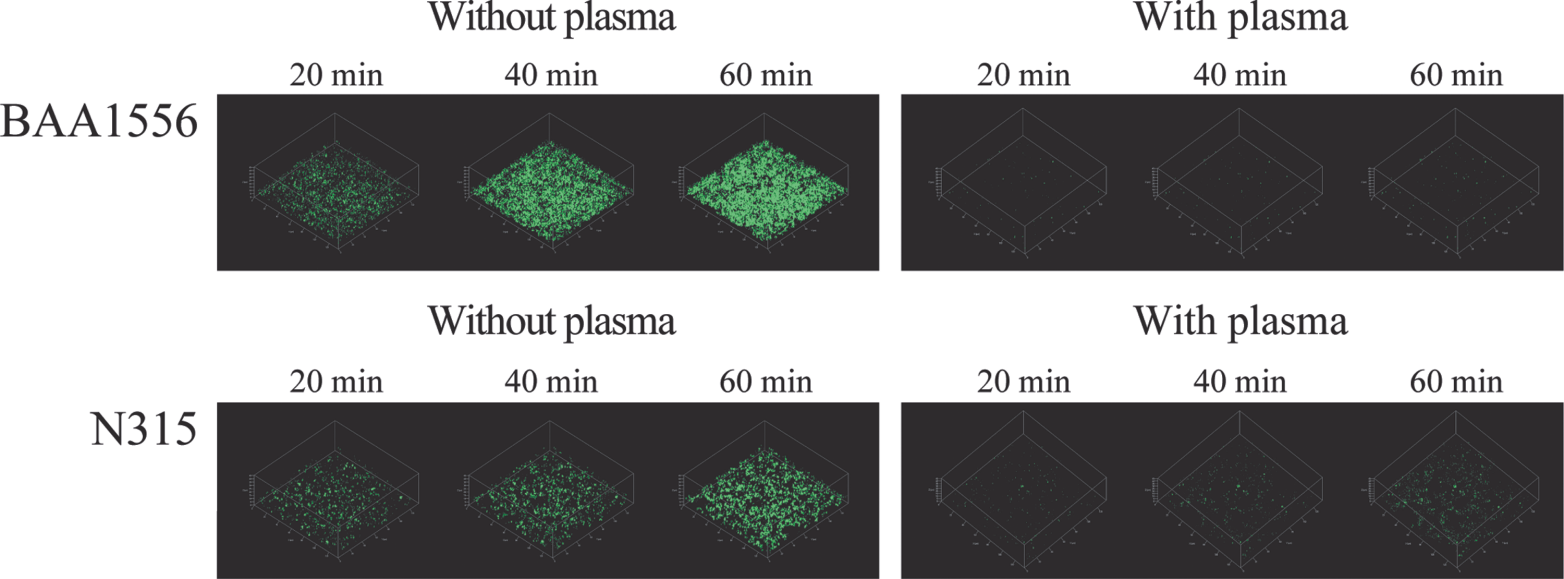
Penetration and accumulation of daptomycin in *Staphylococcus aureus* biofilm over time. Biofilms of BAA1556 and N315 strains were formed during 6 hours incubation with media refreshed every hour, in the presence or absence of 0.78% plasma. Then, green fluorescence-labeled daptomycin was added. Accumulation of daptomycin was compared at 20, 40, and 60 minutes using CLSM.

We used green fluorescence-labeled daptomycin and examined the penetration and accumulation of daptomycin in *S. aureus* biofilms. Since the fluorescence intensity of daptomycin was weaker than that of vancomycin, a higher concentration of daptomycin was used in the experiment. In biofilm formed in the absence of plasma, green fluorescence was visible from 10 to 20 minutes, and after that, the fluorescence intensity increased in a time-dependent manner for both strains of *S. aureus*. Conversely, green fluorescence was hardly detectable in the plasma biofilm, even after 60 minutes of incubation (Fig. 5). These data suggest it delayed penetration and accumulation of daptomycin in plasma biofilm, compared to plasma-free biofilm. Unfortunately, we could not compare the data between vancomycin and daptomycin, because of the differences in the green fluorescence intensities of the antibiotics.

### Effect of antibiotics on the bacterial amount in biofilm formed with or without plasma

**FIG. 6.**
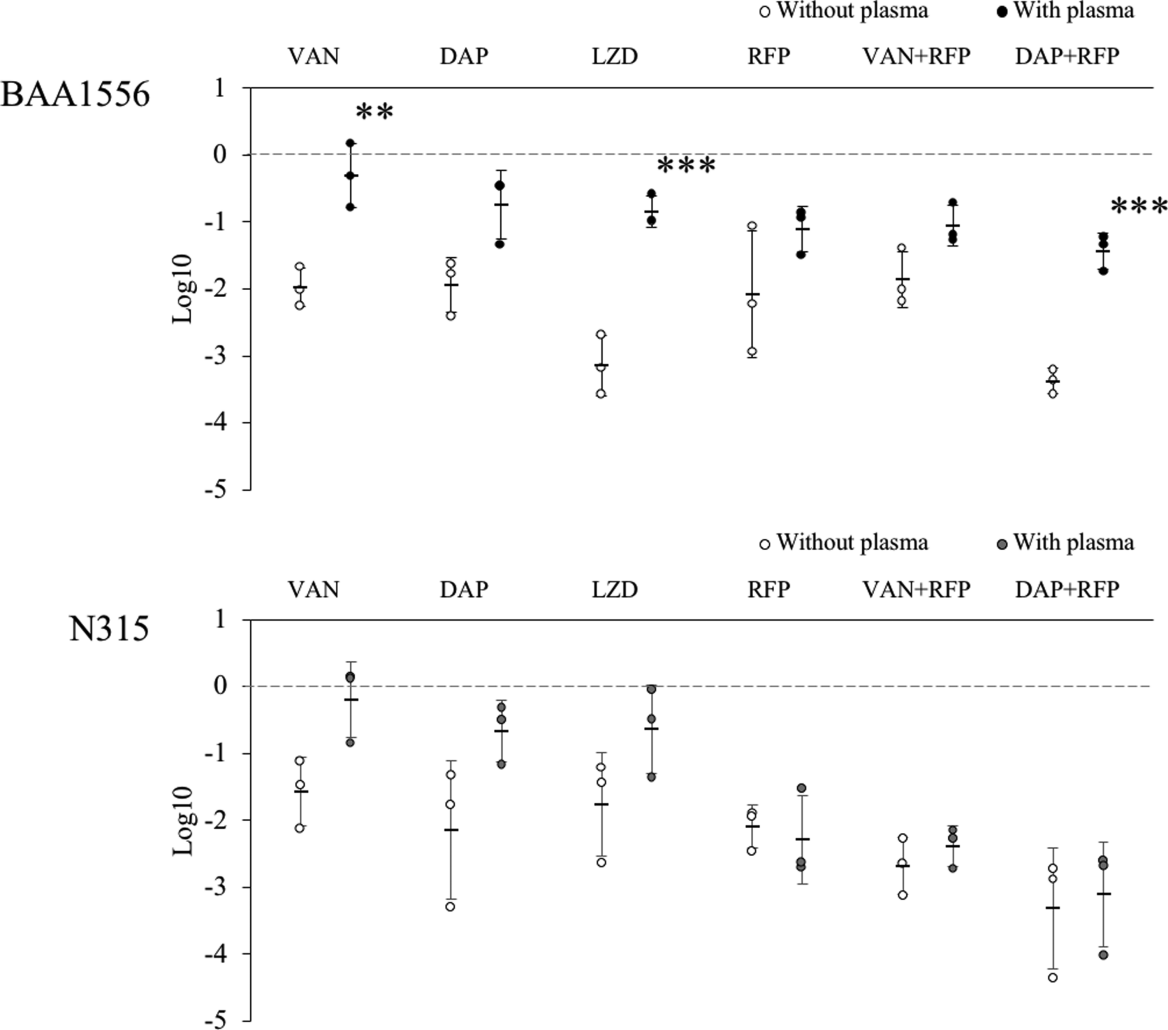
Effect of antibiotics on the bacterial number in biofilm formed with or without plasma. Biofilms of BAA1556 and N315 strains were formed during 6 hours incubation with media refreshed every hour, in the presence or absence of 0.78% plasma. Then, antimethicillin-resistant *Staphylococcus aureus* agents were added at 64 × minimum inhibitory concentration and incubated for an additional 12 hours. Viable bacterial number was determined by plating serial 10-fold dilutions (*n* = 3). The decrease in the number of bacteria is indicated based on the number of bacteria in each control. The bactericidal activity against biofilms made with or without plasma was compared and tested for each antibiotic. The bactericidal activity of single agents of vancomycin or linezolid, or a combination of daptomycin and rifampicin against the plasma-containing biofilm of BAA1556 was significantly reduced. (ANOVA, Tukey's test, **p < 0.01; ***p < 0.001). DAP, daptomycin; LZD, linezolid; RFP, rifampicin; VAN, vancomycin.

We compared the bactericidal activity of antibiotics on biofilm formed with or without plasma. Before treatment, similar bacterial cell counts were recovered from plasma and plasma-free biofilms. This result suggests that the amount of BF formation increased proportionally to the extent of extracellular component accumulation, including the coagulation factor protein, but independently from the number of adhering bacterial cells. Anti-MRSA antibiotics vancomycin, daptomycin, or linezolid, with rifampicin reduced bacterial number in the plasma-free biofilm. However, the plasma biofilms of both strains of *S. aureus* were generally more resistant to the antibiotics, even in combination with rifampicin. For the BAA1556 strain, no condition induced more than two log reduction in bacterial number, whereas the N315 strain showed a significant reduction in bacterial number in plasma biofilm in the presence of rifampicin, vancomycin with rifampicin, and daptomycin with rifampicin (Fig. 6). These results suggest that plasma biofilms are more resistant to the bactericidal activity of the anti-MRSA antibiotics examined. We also performed viability staining to differentiate live and dead cells in the above samples. Some staining results were consistent with the bacterial number, but there were several discrepancies between the staining and bacterial counts (Supplementary Fig. S1).

## Discussion

COCRM method was, for the first time, applied for the observation of *S. aureus* biofilm, which showed the rapid growth of biofilms in the presence of plasma. Delayed antibiotic penetration or accumulation and resistance to the bactericidal activity of anti-MRSA agents were also observed in the plasma biofilms. These results suggest that plasma biofilm is substantially different morphologically and biologically from the plasma-free biofilm.

As shown in Fig. 1, the presence of plasma enhanced biofilm formation at 6 hours in both strains of *S. aureus*, even at a concentration as low as 0.09% plasma. At later time points, the biofilm formation was enhanced for the BAA1556 strain in the presence of 3.12% or more plasma, but not the N315 strain. Conversely, a trend of reduction in biofilm mass was observed for the N315 strain in higher plasma concentrations. Since the crystal violet staining method evaluates the attachment of biofilm to microplate wells, it is likely that detachment of biofilm and new biofilm formation could reflect the total biofilm mass. We, therefore, observed the dispersion or detachment of biofilm in the flow-cell system and through static incubation, especially at higher concentrations of plasma at later time points of incubation (data not shown).

In the flow-cell system observed by COCRM, aggregated biofilm was observed within 3 hours of incubation, which was similar to 12 hours of incubation of plasma biofilm in the static condition. A continuous supply of plasma probably more closely mimics the *in vivo* environment, which could accelerate biofilm formation in the flow-cell system. COCRM is a newly developed technique,^13^ which enabled us to observe the time course of maturation and detachment of biofilms without using fluorescent dyes. Therefore, COCRM could be used as a powerful tool to analyze the mechanism of biofilm formation and to evaluate potential therapeutic strategies in biofilm research.

We observed significant differences between vancomycin and daptomycin accumulation between plasma and plasma-free biofilm. Jefferson *et al.* reported that while vancomycin binds to free-floating bacteria in water within 5 minutes, it took more than 1 hour to bind cells within the deepest layers of a plasma-free biofilm.^16^ In plasma-free biofilm experiments, at therapeutic concentrations of vancomycin, the biofilm matrix was not an obstacle to the diffusion of vancomycin.^17^ However, when human plasma-coated wells were used, increased drug susceptibility was observed transiently in the early phase (≤24 hours), whereas older, dense biofilms exhibited a high level of resistance at later time points (>48 hours).^6^ Cardile *et al.* demonstrated that exposure of *S. aureus* to plasma resulted in a significant increase in microbial surface components, and plasma-augmented biofilms displayed increased tolerance to vancomycin compared to biofilm grown in plasma-free media.^7^ Furthermore, in the mouse model of prosthetic vascular graft infections, daptomycin was ineffective in eradicating biofilms, because the matrix acted as a shield to antibiotic diffusion.^18^ These results suggest that *S. aureus* biofilm-related resistance or tolerance to anti-MRSA agents could be related to multiple factors, such as the presence of plasma, time of incubation, change in bacterial surface structure, and strain differences.

We observed reduced bactericidal activity with the anti-MRSA antibiotics in plasma biofilm, compared to plasma-free biofilm in both strains. Although the amount of biofilm formation increased in the presence of plasma, the number of bacteria did not increase. It is thought that the increase in biofilm formation obtained using plasma components interferes with the activity of the antimicrobial agent. In the N315 strain, the addition of rifampicin increased the bactericidal activity of vancomycin and daptomycin, but its effect was poor with the BAA1556 strain. Although the present study examined two different strains, CA-MRSA (BAA1556) and HA-MRSA (N315), it is necessary to examine more strains for a better understanding of strain differences and resistance mechanisms and to identify effective therapeutic combinations for plasma biofilm.

In this study, we used rabbit plasma to analyze plasma biofilm. However, human plasma should be used to clarify the actual behavior of plasma biofilm in the human body. Furthermore, plasma was used in low concentration (0.39 − 7.14%) because high concentration plasma causes clotting of the medium by coagulase from MRSA, which hinders evaluating the plasma biofilm. However, a significant increase in the amount of biofilm formation was confirmed under these conditions of reduced coagulation factor protein, compared to blood. A coagulation system could be activated *in vivo*, and therefore further studies to investigate conditions closer to the human body are required in the future.

In conclusion, we have reported the effects of plasma on the biofilm formation of CA-MRSA and HA-MRSA. The data show that plasma is a crucial factor in determining not only biofilm mass itself but also the biological nature of the biofilm, including sensitivity to penetration or accumulation of antibiotics and resistance to anti-MRSA drugs. These results further emphasize the importance of plasma biofilms in understanding the hurdles associated with eradication and treatment in bedside settings. Plasma biofilms formed in the flow-cell model could represent a clinically relevant experimental condition, and the application of COCRM facilitates further analysis of biofilm formation mechanisms and allows screening for novel strategies to fight MRSA infections.

## Supplementary Material

Supplemental data
