# Supplementary material for: Morphological and Biological Characteristics of Staphylococcus aureus Biofilm Formed in the Presence of Plasma
Source: Microb Drug Resist. 2019 May 30;25(5):668–76. doi: 10.1089/mdr.2019.0068 (PMC6555173; doi:10.1089/mdr.2019.0068)
Supplement: Supplemental data [file Supp_Fig1.pdf]

Supplementary Data

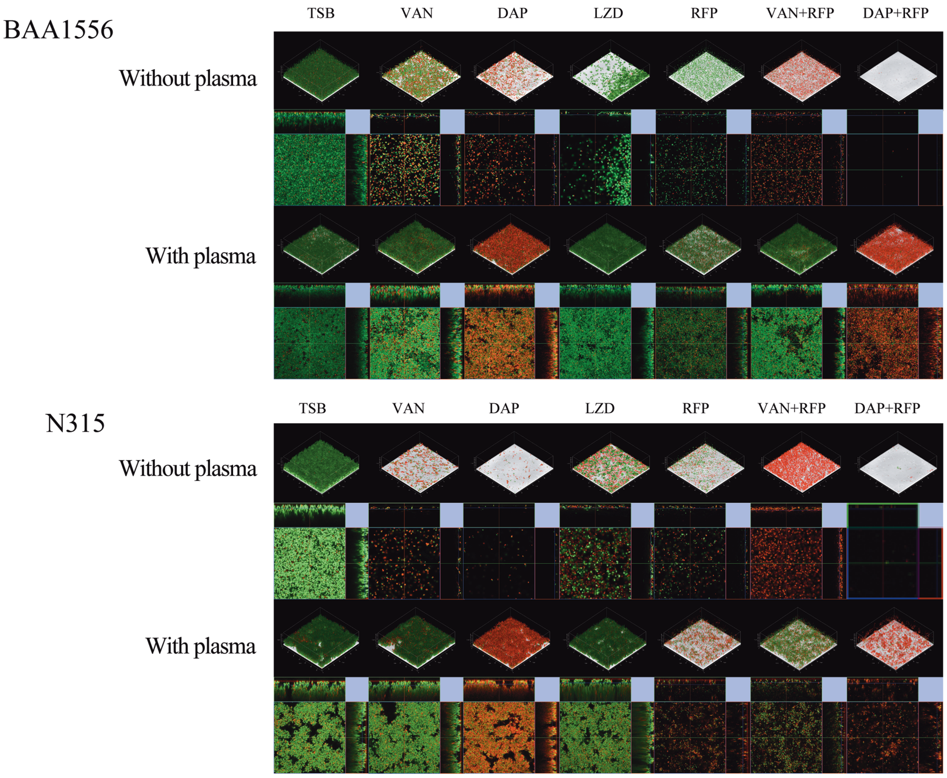

SUPPLEMENTARY FIG. S1. Viability staining of *Staphylococcus aureus* biofilm with or without plasma after treatment with anti-MRSA agents. Biofilms of BAA1556 and N315 strains were formed during 6 hours incubation with media refreshed every hour, in the presence or absence of 0.78% plasma. Then, anti-MRSA agents were added at 64 × minimum inhibitory concentration and incubated for an additional 12 hours. Viable cells were stained *green* with SYTO 9, whereas dead bacteria were stained *red* with propidium iodide. DAP, daptomycin; LZD, linezolid; MRSA, methicillin-resistant *S. aureus*; RFP, rifampicin; VAN, vancomycin.
